# Chinese society of cardiology expert consensus statement on the diagnosis and treatment of adult fulminant myocarditis

**DOI:** 10.1007/s11427-018-9385-3

**Published:** 2018-12-03

**Authors:** Daowen Wang, Sheng Li, Jiangang Jiang, Jiangtao Yan, Chunxia Zhao, Yan Wang, Yexin Ma, Hesong Zeng, Xiaomei Guo, Hong Wang, Jiarong Tang, Houjuan Zuo, Li Lin, Guanglin Cui, Section of Precision Medicine Group of Chinese Society of Cardiology, Editorial Board of Chinese Journal of Cardiology, Working Group of Adult Fulminant Myocarditis

**Affiliations:** Division of Cardiology, Department of Internal Medicine, Tongji Hospital, Tongji Medical College, Huazhong University of Science & Technology, Wuhan, 430030 China

**Keywords:** adult fulminant myocarditis, diagnosis and treatment, expert consensus, life support-based comprehensive treatment regimen, cardiogenic shock, mechanical circulatory support

## Abstract

Fulminant myocarditis is primarily caused by infection with any number of a variety of viruses. It arises quickly, progresses rapidly, and may lead to severe heart failure or circulatory failure presenting as rapid-onset hypotension and cardiogenic shock, with mortality rates as high as 50%–70%. Most importantly, there are no treatment options, guidelines or an expert consensus statement. Here, we provide the first expert consensus, the Chinese Society of Cardiology Expert Consensus Statement on the Diagnosis and Treatment of Fulminant Myocarditis, based on data from our recent clinical trial (NCT03268642). In this statement, we describe the clinical features and diagnostic criteria of fulminant myocarditis, and importantly, for the first time, we describe a new treatment regimen termed life support-based comprehensive treatment regimen. The core content of this treatment regimen includes (i) mechanical life support (applications of mechanical respirators and circulatory support systems, including intraaortic balloon pump and extracorporeal membrane oxygenation, (ii) immunological modulation by using sufficient doses of glucocorticoid, immunoglobulin and (iii) antiviral reagents using neuraminidase inhibitor. The proper application of this treatment regimen may and has helped to save the lives of many patients with fulminant myocarditis.

Myocarditis refers to inflammatory injuries caused by various pathogens that can finally result in dysfunction of the heart, such as decreased systolic or diastolic function and arrhythmias (Caforio et al., 2013; Caforio et al., 2017; Fung et al., 2016; Lazaros et al., 2017; Luyt et al., 2016; Pollack et al., 2015). The common causes can be classified into three classes: infection; autoimmune disease (giant cell myocarditis, sarcoidosis, systemic lupus erythematosus, Churg-Strauss syndrome, and mucocutaneous lymph node syndrome) and drug hypersensitivity; and poisons or toxic drug effects. Infections cause the majority of cases of myocarditis with the most common pathogens being viruses, including enteroviruses (especially Coxsackie B virus), adenovirus, cytomegalovirus, Epstein-Barr virus (EBV), and influenza virus. There are usually three clinical stages of myocarditis: the acute phase, subacute phase, and chronic phase. The acute phase lasts for 3–5 days, during which active viral replication and invasion severely damage the myocardium. Immunological reaction plays a major role in the subacute phase, and only a few patients eventually enter the chronic phase. In this phase, patients may suffer from chronic, persistent, but sudden aggravated inflammatory reactions as well as decreased contractility, myocardial fibrosis, and cardiomegaly (Caforio et al., 2013; Fung et al., 2016; Pollack et al., 2015). Myocarditis has a wide range of clinical manifestations, ranging from slight squeezing chest depression after activity, to acute left heart failure, cardiogenic shock, and even sudden death. Therefore, treatment must be personalized according to the severity of the condition.

Fulminant myocarditis is the most severe type of myocarditis and is characterized by sudden occurrence; rapid progress; hemodynamic dysfunction, such as pump failure and circulation failure that can develop very quickly; and respiratory, liver, or kidney failure which can occur simultaneously. All these complications can result in a high mortality rate at the early stage of the disease (Ginsberg and Parrillo, 2013; Gupta et al., 2008; Maisch et al., 2014; McCarthy et al., 2000). Viral infection may be the most common cause of fulminant myocarditis, while other causes such as autoimmune diseases, hypersensitivity, or drug-induced fulminant myocarditis are rare. However, no distinguishing histological and pathological difference can be found between fulminant myocarditis and common viral myocarditis. Thus, fulminant myocarditis can be considered to be a clinical rather than a pathophysiological diagnosis. It is thought that when acute myocarditis shows a rapid onset and dramatically accelerated progress and a situation arises where patients with severe heart failure, hypotension, or cardiogenic shock must receive positive inotropic drugs and vasoactive agents or mechanical circulation assist device to maintain or save life, the condition may be called fulminant myocarditis. It is noteworthy that although fulminant myocarditis has a high early mortality, if patients can survive through the acute phase, they will have a good long-term prognosis, similar to healthy persons (McCarthy et al., 2000). An 11-year follow-up study revealed that patients suffering from fulminant myocarditis have higher survival rates than those with common myocarditis (93% vs. 45%), and there was no difference in long-term survival between patients with fulminant myocarditis and healthy people (McCarthy et al., 2000). This suggests that patients who suffer from fulminant myocarditis are often healthy young adults. Besides, fulminant myocarditis has a higher incidence rate during the winter and spring seasons. Although fulminant myocarditis has a higher prevalence rate among young adults and children, it can attack people at any age with no difference between genders. Prolonged tiredness or exhaustion may contribute to its occurrence. Therefore, if a patient is suspected as having fulminant myocarditis, great care must be taken to employ a multidisciplinary approach to reach a quick diagnosis and start treatment promptly to give patients the best possible chance of surviving through the dangerous acute phase.

Due to the limited number of randomized trials investigating treatment of fulminant myocarditis, to date, there are no guidelines or even expert consensus statement on its diagnosis and treatment. However, given the very high mortality and the severity of fulminant myocarditis, we have decided to comprehensively review existing publications and summarize our treatment experience in the last three years from registered open clinical trial in order to provide to practicing physicians an expert consensus on its clinical diagnosis and treatment. We hope that this will improve treatment standards and greatly improve survival rates. Since viral infection is the dominant cause of fulminant myocarditis, and treatment of fulminant myocarditis resulting from other causes is similar except for antiviral use, this consensus statement is focused on viral fulminant myocarditis.

## Pathophysiology of fulminant myocarditis

The fundamental cause and pathophysiological mechanisms underlying fulminant myocarditis are similar to that of acute, non-fulminant myocarditis. It is thought that virus infection is a major cause of acute myocarditis (Caforio et al., 2013; Maisch et al., 2014; Pollack et al., 2015), but due to the limitations of detection technology, viral genes can be detected in only 10%–20% of patients' myocardial samples. Among detectable viruses, the most common are Coxsackie B virus, adenovirus, and influenza virus. Contemporary rates of infection with influenza virus, especially highly-pathogenic strains, are very high. The pathophysiology of myocardial injury includes direct damage by the virus and damage caused by the immune reaction and cytokines. Direct damage is more common in newborns, while, in contrast, immunogenic injury is the major factor in adults.

### *Mechanisms of myocardial injury*

(1) Direct injury: Intracellular viral replication in the myocardium and other tissues, results in degeneration, necrosis, and dysfunction. When the virus is released from the cell, its progeny continues to infect other myocardial cells or tissues. Cytokines will also be released from myocardium and damage other cells and organs.

(2) Immunogenic injury: On the one hand, cytokines released from cells injured by viral infection will cause inflammatory edema. On the other hand, due to the recruitment effects of cytokines, inflammatory cells such as mononuclear macrophages, lymphocytes, and neutrophils will infiltrate the matrix. The myocardium thus is damaged as a result of the ensuing cytotoxic and antigen-antibody reaction. In response to viral infection, the cellular and humoral immune response will stimulate infiltrative inflammatory and tissue cells to release a substantial cascade of cytokines, such as interleukin-1 or -6 (IL-1; IL-6), endothelial adhesion molecules, and tumor necrosis factor α (TNFα) among others. These cytokines will damage the myocardium as well as other tissues. In addition, the cytokines activate leukocytes to form complexes with platelets, which will cause the formation of thrombus, diffuse intravascular coagulation, and stimulate leukocyte migration to tissues.

Direct viral injury of the myocardium in fulminant myocarditis is indeed severe; however, abnormal activation of the immune system and excessive macrophage polarization and accumulation in tissues or organs can cause indirect injury and is an important pathophysiological of rapid progression. It must be kept in mind that fulminant myocarditis does not only center around myocardial injury; viral invasion, cytokine release, and the immune reaction but can also cause multiple organ lesions throughout the body. Fulminant myocarditis is a systemic disease that mainly affects the myocardium and cardiac reactions are the most noticeable. The heart will suffer predominantly, and this is the cause of hemodynamic dysfunction and death. Therefore, pump dysfunction due to heart lesions determines the severity and high mortality of fulminant myocarditis. The use of a cardiac pump and mechanical circulation support, such as intra-aortic balloons (IABP), extracorporeal membrane oxygenation (ECMO), and left ventricular assist devices (LVAD), decides the final outcome in patients. Meanwhile, protective and replacement treatment targeted at other impaired organs, such as hemodialysis and respirator assisted ventilation, are important in helping patients survive through the acute phase of fulminant myocarditis.

Myocardial edema, apoptosis, necrosis, and inflammatory cell infiltration are the major pathological changes observed in fulminant myocarditis. According to the different types of infiltrating cells, we can classify fulminant myocarditis into neutrophilic, lymphatic, acidophilic, or giant cell myocarditis. It is thought that extensive myocardial necrosis and over 50 infiltrated inflammatory cells per millimeter squared of myocardium can be observed in fulminant myocarditis. However, the relationship between pathological changes and severity of clinic manifestation is not linear. Some patients may have severe clinical manifestations but do not display corresponding pathological changes. Therefore, fulminant myocarditis is more likely a clinical diagnosis. In addition, another important feature of fulminant myocarditis is that while it exhibits a quite severe manifestation during the acute phase, patients who successfully survive this phase will have a good outcome. This is perhaps the most important difference between fulminant and acute myocarditis.

## Clinical evaluation of fulminant myocarditis

Patients with myocarditis have different degrees of manifestations, from slight chest pain, palpitation, and transient electrocardiogram (ECG) changes to life-threatening cardiac shock and severe arrhythmia. Fulminant myocarditis, however, is the most severe type of myocarditis. It arises quickly, progresses rapidly, and may lead to serious heart failure or circulatory failure presenting as hypotension and cardiogenic shock in a short time. Different types of arrhythmia may also occur and threaten patients' lives. Respiratory, liver, and kidney failure may further worsen patients' conditions. In general, vasoactive agents or positive inotropic drugs must be administered and mechanical circulation assist device or respiratory support must be employed to maintain adequate circulation. Although fulminant myocarditis has a higher prevalence rate among young adults and children, it can attack people at any age with no difference between genders. Fulminant myocarditis has a higher incidence rate during the winter and spring seasons. Prolonged tiredness or exhaustion may contribute to its occurrence.

### *Symptoms*

*Premonitory symptoms of viral infection*

Patients may have fever, weakness, nasal obstruction, pharyngalgia, cough, and diarrhea in the early stages. It must be noted that there is no unique symptom profile at the very stages of the disease, and many patients may simply develop a slight fever, feel weak, have significantly reduced appetite, or mild diarrhea. These symptoms can last for 3–5 days or even longer, and are usually neglected by patients and even by physicians. However, they are important clues toward a diagnosis of myocarditis, and therefore it is quite important to take a detailed history.

*Symptoms of cardiac injury*

Several days or 1–3 weeks after the premonitory symptoms of viral infection, patients may come to consult the physician due to panting, dyspnea, chest distress or pain, palpitation, dizziness, extreme weakness, and obvious loss of appetite. Data from Europe reveals that 72% patients have dyspnea, 32% have chest pain, and 18% have arrhythmias (Hufnagel et al., 2000). Data collected from Tongji Hospital at Wuhan shows that 90% of patients are transferred because of dyspnea, and 10% for syncope or after-cardiac-pulmonary resuscitation.

*Hemodynamic dysfunction*

It is one of the most important features of fulminant myocarditis. Some patients may rapidly develop acute left heart failure or cardiac shock, and have symptoms of passive pulmonary congestion and shock-like severe dyspnea, orthopnea, whooping pink foam, serious anxiety, hyperhidrosis, oliguria, or anuria. Symptoms of shock such as skin clamminess, pallor, cyanosis, patch pattern changes of the skin, and even disturbances of consciousness can also be present. A few patients will have fainting episodes, syncope, or sudden death. Importantly, among the three basic determinants of cardiac output (CO; myocardium contractility, preload, and afterload), failure of heart pump function is responsible for hypotension in patients with fulminant myocarditis (e.g., those with cardiogenic shock) while blood volume and vascular resistance are only minor factors. Since patients with fulminant myocarditis usually have no underlying cardiac diseases, their heart sizes are usually normal, and they simply suffer from a marked diffuse decrease in myocardial contractility and a reduction in the cardiac ejection fraction. The importance of the severity of myocardium injury to hypotension and cardiac shock is often ignored. Since most patients have normal heart sizes and the disease progresses very rapidly, the heart is unable to compensate for the injuries, which further aggravates cardiac dysfunction.

*Symptoms of injury to other organs*

Fulminant myocarditis can also involve other organs, causing dysfunction or even failure. This includes liver dysfunction (glutamic-oxalacetic transaminase levels can increase to as high as 10,000–20,000 U $L^{-1}$, and enzyme-bilirubin separation further complicates matters), kidney injury (increase of serum creatinine level, oliguria, or even anuria), blood coagulation disorders (hemorrhage or diffuse intravascular coagulation), and respiratory injury (pulmonary injury, infection, hypoxemia, and even acute respiratory distress syndrome (ARDS)). Dysfunctions of these organs are partly secondary to cardiac injury (shock), but viral invasion and immunological injury play important roles. These injuries may lead to a rapid deterioration of the patients' condition. Some patients have severe pulmonary injury, and as a result are diagnosed with severe pneumonia due to hypoxemia or dyspnea, and the underlying fulminant myocarditis is overlooked.

## Signs

*Vital signs*

Abnormal blood pressure, respiration, and heart rate often suggest hemodynamic abnormalities. The most obvious features of fulminant myocarditis that can indicate its severity are as follows:

(1) Body temperature: Some patients may have an increase in body temperature due to the viral infection. If there is a concomitant pulmonary infection or other bacterial, the body temperature may reach up to 39°C. Some patients may have hypothermia (body temperature lower than 36°C), which is an indicator of disease severity.

(2) Blood pressure: Hypotension is very frequent because of severe cardiac dysfunction and abnormalities of vasoactivity due to the global toxic reaction. Patients with particularly severe disease may have an undetectable blood pressure.

(3) Respiration: Abnormalities witnessed include tachypnea (more than 30 breaths per minute (bpm)), respiratory depression (may be less than 10 bpm in serious cases), and artery blood oxygen saturation disturbances (often lower than 90%, may reach as low as 40%–50%).

(4) Heart rate: Tachycardia (often faster than 120 bpm) or bradycardia (can be slower than 50 bpm). Sinus tachycardia is one of the most obvious features of fulminant myocarditis. The heart rate is usually over 100 bpm, and may be higher than 160 bpm. The mismatch between increased heart rate and body temperature (often >10 bpm per elevation of one degree centigrade) is not very specific, but it is an important clue toward making the diagnosis of fulminant myocarditis, and attention should be paid to it. Besides sinus tachycardia, different arrhythmias may also occur, including ventricular or supraventricular premature contractions, ventricular or supraventricular tachycardia, and ventricular fibrillation among others. In addition, injuries to the heart's conduction system can result in bradycardia, sinus arrest, and conduction blocks. Fast ventricular tachycardia, ventricular fibrillation, sinus arrest, and third degree atrioventricular block can cause Adams-Stokes syndrome and threaten patients' lives.

*Signs related to heart*

The heart border is usually normal. The cardiac beat can weaken due to reduction in cardiac contractility. Heart sounds are blunt, and the third heart sound or third heart sound gallop can usually be heard. There are moist rales in the lung because of left heart dysfunction or complicated pneumonia. Right heart failure is rarely present.

*Other signs*

A wet and cold body skin texture, skin patch pattern changes, and poor peripheral circulation can be observed when shock occurs. Hypoperfusion and injury to the brain can cause irritability, consciousness disturbance, or even coma. Liver injury can result in jaundice and abnormal liver function. Skin ecchymosis can be observed because of coagulation disturbances and microcirculation dysfunction.

### *Auxiliary examinations*

*Laboratory tests*

(1) Myocardial injury markers and myocardial enzymes: There is a dramatic elevation in the levels of serum myocardial injury marker troponin I or T (cTnI and cTnT); or the myocardial enzymes, creatine kinase (CK) or creatine kinase isoenzyme, lactic dehydrogenase (LDH), aspartate aminotransferase (AST); and myoglobin. CTnI or cTnT is the most sensitive and specific marker. Based on natural history, the differences in myocardial enzymes changes between fulminant myocarditis and myocardial infarction are: (i) There is no obvious enzyme peak in fulminant myocarditis, which indicates that it is a progressive change; (ii) The increase of enzymes lasts for a long period, which indicates persistent and progressive injuries to the myocardium, and it often predicts a poor prognosis. However, efficient therapy will change the natural history.

(2) Brain natriuretic peptide (BNP or NT-BNP): There is usually a dramatic rise in BNP levels, which represents severe cardiac injury. It is an important index to evaluate the severity of heart dysfunction and helps judge the development and outcome of the disease. BNP or NT-BNP is also a valuable index for the differential diagnosis of fulminant myocarditis and severe pneumonia. Attention must be paid to the fact that the marked rise in BNP levels after myocardial injury is usually delayed. Therefore, patients who have normal BNP or a slight increase in BNP at the very beginning of the outbreak of fulminant myocarditis need reexamination within a short period.

(3) Routine blood tests: Neutrophil number and ratio do not rise at first, but will increase 2–3 days later. Neutrophils will rise when a patient's condition is complicated with bacterial infection. A persistent decrease in neutrophil numbers indicates poor prognosis. The number of monocytes will increase. Severe toxemia always depletes platelets. A sustained decrease in platelet numbers indicates myelosuppression and is a predictor of poor prognosis similar to a marked decrease in neutrophil number. Leukocytes will rise when myocarditis is complicated by bacteria infection. When infection takes place, erythrocyte sedimentation rate and C-reactive protein levels may rise, but they are not specific signs. Cytokine levels, including TNF, IL-10, IL-6, IL-1, and endothelial adhesion molecules will rise. Some patients with fulminant myocarditis will suffer from multiple organ failure, especially liver and kidney injury, as a result of viral infection, immunological injury, and shock.

*ECG*

ECGs are sensitive but not particular to the diagnosis of fulminant myocarditis. Therefore, performing repeated ECGs and comparing the differences are recommended. Sinus tachycardia is the most common finding. Frequent atrial or ventricular premature beat is one of the reasons of hospitalization. Paroxysmal ventricular tachycardia may be frequently found when patients are monitored. The occurrence of bundle branch block or atrioventricular block is a predictor of poor prognosis. Low limb lead voltage, especially precordial leads, is a sign of diffuse and severe myocardial injury. Changes in ST-T, which represents abnormal myocardial repolarization, are very common. Some patients may have ECG findings similar to typical acute myocardial infarction (AMI), such as lead-specific arched ST-segment elevation, and it is difficult to differentiate them using ECG alone. Ventricular fibrillation is rare, but is a major reason for sudden death or syncope. Attention should be paid to the fact that ECGs can change from minute to minute; thus, continuous monitoring is required. All patients with fulminant myocarditis should be monitored with a Holter.

*Chest X-ray and computed tomography*

Cardiac silhouette is normal or just slightly enlarge in most patients. However, enhanced hilar vascular shadows, increased superior pulmonary shadows, and fuzzy lung fields, which indicate pulmonary congestion or pulmonary edema caused by left heart dysfunction, are frequently observed. Butterfly-like lung hila and a large area of confluent shadow in lung fields can be seen when acute alveolar pulmonary edema occurs. The so-called "white lung" may be seen when the condition is complicated with viral pneumonia and pulmonary congestion caused by serious heart failure, and as a result, the entire lung field is infiltrated by inflammatory cells that soon progress into a severe diffuse lesion. At that time, the patient will show signs of ARDS and hypoxemia. Pleural effusion and inter-lobe pleural thickening can be observed in some patients.

*Echocardiography*

Echocardiography examination is of great value in the diagnosis and follow-up of fulminant myocarditis. The following changes can be observed:

(1) Diffuse ventricular wall motion decrease: Apparent as a diffuse peristaltic beat, this is a result of decreased myocardial contractility caused by severe diffuse inflammation. It is a rapid change and quickly progresses at an early stage.

(2) Marked reduction in myocardial contraction capability: A dramatic decrease in cardiac ejection fraction can be seen, where it may fall to as low as 10%. The ratio between early mitral inflow velocity and mitral annular early diastolic velocity (E/e') will also rise, indicating reduction in diastolic function, but it will return to normal within several days as the patient improves.

(3) Changes in cardiac chamber sizes: Most patients will have normal sizes, a few patients will have slight increases, and only rarely will obviously enlarged left ventricular cavities be seen.

(4) The thickness of the interventricular septum or ventricular wall can increase slightly as a result of myocardial inflammatory edema.

(5) Ventricular wall motion can show regional abnormalities because of asymmetry or non-uniform myocardial inflammation and damage.

All these changes can return to normal after 10 days or slightly longer with effective treatments (Felker et al., 2000). Another value of ECG is that it can help rule out other diseases like valve disease and hypertrophic or restrictive cardiomyopathy. A typical regional reduction in left ventricular wall motion helps diagnose AMI. Pericardial effusion signifies inflammation of the pericardium. Furthermore, ECGs are easily performed, and therefore, it is suggested that they are performed every day or several times per day in order to provide a dynamic evaluation of cardiac function.

*Coronary arteriography*

Some myocarditis patients, especially those who have pericardial inflammation, usually present to the hospital because of acute chest pain or chest discomfort. The ECG may show ST-segment elevation in neighboring leads and myocardial biomarkers may rise, making it difficult to differentiate myocarditis from AMI. Coronary arteriography is recommended to reach a quick diagnosis since treatment for these two diseases are very different. Although coronary arteriography carries a risk of death, retrospective analysis of contemporary data shows no increase in the death rate with urgent arteriography. It is worth noting that as little contrast agent as possible should be used to avoid its inhibitory effect on cardiac contraction.

*Invasive hemodynamic monitoring*

It is suggested that float catheters be used to monitor right atrial, right ventricular, pulmonary artery, and pulmonary capillary wedge pressure. Alternatively, pulse index continuous cardiac output (PICCO) may be used to monitor pressure. Routine invasive hemodynamic monitoring is recommended to judge the condition of patients and their response to treatments.

*Cardiac magnetic resonance*

Cardiac magnetic resonance (CMR) has been increasingly applied to the diagnosis of myocarditis. Not only can CMR show the physical structure of the heart, it can also help assess cardiac functions. More importantly, pathological changes in cardiac tissues can be observed directly via CMR, which can provide various imaging evidence of edema, congestion, necrosis, or fibrosis of the myocardium. The value of CMR to the diagnosis of myocarditis has been increasingly recognized in recent years, but due to the limitations posed by the nature of the illness itself and the need for urgent treatment, as well as the inconvenience of CMR, it is not recommended for clinical use in fulminant myocarditis. CMR should be considered only when the patient is stable and it is difficult to make final diagnosis.

*Percutaneous endomyocardial biopsy*

It is not recommended to perform percutaneous endomyocardial biopsy during the acute phase of fulminant myocarditis. During this phase, patients are in very poor condition and biopsy can only provide limited guidance to clinical diagnosis and does not provide urgent support and life-saving treatment. However, it is still considered to be the golden standard of diagnosis. Therefore, percutaneous endomyocardial biopsy may be performed when the patients start improving to help determine the responsible pathogen and aid in research on the pathophysiological mechanisms of fulminant myocarditis (Caforio et al., 2013).

*Detection of pathogens*

Viral myocarditis is usually caused by respiratory viruses or enteroviruses, the most common being Coxsackie B virus, and detection of IgM antibody may help early diagnosis. Meta genome sequencing and target gene sequencing can help to identify pathogens.

The recommendations on auxiliary examinations used to help in diagnosing myocarditis/fulminant myocarditis and their sequence of use are summarized in Table 1.

## Diagnosis

Fulminant myocarditis is usually defined as myocardial inflammatory disease with a rapid outbreak complicated with severe hemodynamic dysfunction. It is more likely to be a clinical diagnosis rather than a histological or pathological diagnosis. Therefore, the clinical state as well as laboratory and imaging tests should all be taken into full consideration before confirming the diagnosis. Usually, the clinical diagnosis of fulminant myocarditis can be made when patients have following signs: sudden attack, obvious premonitory symptoms of viral infection (especially severe fatigue and poor appetite), rapid emerging severe hemodynamic dysfunction, serious myocardial injuries, and diffuse ventricular wall motion decrease (Sun et al., 2017).

### ***Differential diagnosis***

Since fulminant myocarditis affects multiple organs and systems, has variable and severe clinical manifestations, and progresses rapidly, some examinations should be performed to exclude other diseases such as cardiac diseases (acute attack of coronary heart disease, myocarditis, or valvular diseases) or diseases that have similar clinical manifestations.

**Table 1** Recommendations of auxiliary examinations

| Auxiliary tests | Recommendations |
|---|---|
| Laboratory examinations | 1. All the patients who are clinically suspected of fulminant myocarditis must be tested for biomarkers of myocardium injury. It is an important index to evaluate the injury to myocardium and the effectiveness of treatment.<br>2. All the patients who are clinically suspected of fulminant myocarditis must be tested for BNP or NT-BNP and be monitored dynamically. It is an important index to evaluate the injury to myocardium and the effectiveness of treatment.<br>3. Blood routine and arterial blood gas analysis, blood lactic acid, electrolyte test, liver and kidney function test are recommended. Biomarkers of inflammation like ESR or CRP is also recommended.<br>4. Test of auto-antibody to myocardium can be considered when it is available. |
| ECG | 5. All the patients who are clinically suspected of fulminant myocarditis must receive 12 or 18 lead ECG and be monitored dynamically. |
| Chest X ray/CT | 6. All the patients who are clinically suspected of fulminant myocarditis must receive chest X-ray examination. Patients with unstable hemodynamics or cannot be moved can do bedside X-ray. Chest CT can be tested when the state is stable. Patients with positive findings or severe state should be tested dynamically. |
| Echocardiogram | 7. All the patients who are clinically suspected of fulminant myocarditis must receive echocardiogram and be followed up at bedside.<br>8. Echocardiogram should be carried out at bedside several times a day to help monitor change of cardiac function, observe progress of illness and evaluate prognosis. |
| Coronary arteriography | 9. Immediate coronary arteriography should be carried out to differentiate myocarditis from acute infarction when patients have changes of ECG ST segment, especially for old patients. |
| Invasive hemodynamic monitoring | 10. PICCO or invasive intraartery hemodynamics monitoring should be taken to evaluate the state of illness and judge effects of treatments. |
| Cardiac magnetic resonance/CMR | 11. It can be carried out when hemodynamics of clinically suspected patients is stable.<br>12. It can provide evidence in a noninvasive way, and is possible to substitute percutaneous endomyocardial biopsy. |
| Percutaneous endomyocardial biopsy | 13. It can be considered when patients are clinically suspected of fulminant myocarditis.<br>14. It is still considered as the golden standard of diagnosis of myocarditis.<br>15. It should be taken when patients are clinically suspected of special types like giant cell myocarditis to guide clinical treatment. |
| Detection of pathogens | 16. Tests of serum special antibodies can help early diagnosis.<br>17. Viral gene detection can be done to help identify pathogens if it is available. |

(1) Coronary heart disease: A large area of AMI can cause pulmonary congestion and subsequently lead to circulation failure, shock, and rise of myocardial enzymes and must be distinguished from fulminant myocarditis. Obvious regional motion abnormalities in the ventricle wall can be observed using an echocardiogram. The most important and useful test is coronary arteriography, which is able to differentiate between these two diseases immediately.

(2) Viral pneumonia: Severe viral pneumonia complicated with septic shock can also cause a transient rise of myocardial biomarkers or enzymes. However, these enzymes will return to normal quickly after the improvement of shock and oxyhemoglobin saturation.

(3) Sepsis myocarditis: Severe bacterial infection can damage the myocardium and induce toxic myocarditis. As a result, it can aggravate infectious shock and depress heart function. Previous infection foci, early rise of peripheral blood leukocytes, and other global symptoms can help in diagnosis.

(4) Tako-Tsubo syndrome: Also known as apical ballooning syndrome. It is especially prevalent in post-menopausal women. Chest pain, changes of ST-T segment, and a rise in serum myocardial biomarkers or enzymes indicate disease onset. This syndrome is often triggered by intense stressful events and commonly occurs in post-menopausal women. Left ventriculography can find diffuse motion reduction or regional ventricular wall motion abnormalities, whose area is larger than that supplied by a single coronary artery. The most common lesion location is at the apex, which has a representative "octopus-basket-like change." Coronary arteriography may be negative or find mild coronary artery atherosclerosis. Tako-Tsubo syndrome usually needs only supportive treatment and left ventricular function can quickly recover.

(5) Common myocarditis: Compared with fulminant myocarditis, common myocarditis is characterized by premonitory history of infection, sudden attack, rapid progress, poor patient condition, obvious myocardial injury, quick recovery after appropriate treatment, and good long-term outcome. On the other hand, acute myocarditis does not have these features, and it is possible that the state of illness can be prolonged and progress to chronic or persistent myocarditis or cardiomyopathy (Felker et al., 2000).

(6) Non-viral fulminant myocarditis: Non-viral fulminant myocarditis includes acute fulminant myocarditis caused by autoimmune disease, toxic drug effects, or drug allergies such as penicillin-induced fulminant myocarditis. Patients have no clear sign of viral infection, but have a history of auto-immune disease, usage of toxic drugs, especially anti-neoplastic drugs, or drug allergies. Non-viral fulminant myocarditis has the rapid progress and severe manifestation seen in viral fulminant myocarditis. Treatment is similar to viral fulminant myocarditis, except for the antiviral drugs.

## Treatments

Since fulminant myocarditis has a sudden onset, rapid progress, and high mortality at its early stage, careful, deliberate treatment is always essential. All possible treatments should be employed to save lives. Based on our specialists' experiences and the results of our clinical trial, this consensus statement proposes a "Life Support-Based Comprehensive Treatment Regime." Once fulminant myocarditis is suspected, active integrated treatment should be started as soon as possible. This regimen includes strict bed rest and monitoring of hemodynamics, cardiac electric activity, and blood oxygenation saturation, as well as nutritional support and drug administration including anti-inflammatories, anti-virals, immune modulation using large doses of glucocorticoids and intravenous immunoglobulin (IVIG). Plasma or blood purification and life-support techniques (including IABP, ECMO, assisted mechanical ventilation, and temporary pacemaker implantation if necessary) should be employed if needed. Heart transplantation can be considered when therapy fails.

### *Close monitoring*

Recommendation: All fulminant myocarditis patients should be closely monitored.

Patients should be transferred to hospitals with critical care units (CCU) to receive proper respiratory and circulatory monitoring and life support. Monitoring should include:

(1) Water intake and excretion should be recorded every 1–2 h to provide guidance on the management of fluids and assessments of the state of the patient.

(2) ECG, oxyhemoglobin saturation, and blood pressure.

(3) Changes in laboratory test results such as routine blood tests, cardiac biomarker (cTnI and BNP or NT-proBNP) levels, liver and kidney functions, electrolyte levels, coagulation functions, blood lactic acid levels, and blood gas analysis.

(4) Bedside chest radiograph may be repeated according to the condition of patients with obvious pulmonary changes and hydrothorax.

(5) Bedside echocardiography. Since the disease progresses rapidly, echocardiography can be performed several times a day to evaluate the sizes of cardiac chambers, the changes of ventricular wall motion, and the ejection fraction.

(6) Invasive hemodynamic monitoring, including invasive artery blood pressure, central venous pressure, pulmonary capillary wedge pressure, or PICCO.

### *Active symptomatic and general treatment and supportive treatment*

Recommendation: All fulminant myocarditis patients should receive active symptomatic and general management and supportive treatment.

It is of great importance for patients to receive both symptomatic and supportive treatment. These treatments often include:

(1) Absolute bed rest and reducing visits and disturbances as much as possible to avoid emotion stimulation.

(2) Once patients are able to eat, light, digestive, and nutritious food should be given in small amounts but more frequently.

(3) Oxygen supplied through a nasal catheter, mask, or continuous positive airway pressure oxygen supply. The latter helps reduce cardiac load.

(4) Modulation of myocardial metabolism with administration of phosphocreatine or coenzyme Q10. Trimetazidine can be beneficial to improve cardiac function (Chen et al., 2016).

(5) Water-soluble and lipid-soluble vitamin supplementation.

(6) Administration of sufficient fluids and electrolytes. The amount of liquid infusion must be determined according to the patient's fluid excretion and should be distributed evenly. Fast infusion and excretion of liquid must be avoided for fear of inducing shock or acute heart failure.

(7) Proton pump inhibitors can be administered to protect the gastric mucosa from stress ulcers and digestive tract hemorrhage, especially that caused by glucocorticoids.

(8) Physical cooling or glucocorticoids are recommended when the patient is highly febrile, but non-steroidal anti-inflammatory drugs are not recommended.

### *Anti-viral therapy*

Recommendation: All patients with fulminant myocarditis should receive anti-viral therapy as early as possible.

Theoretically, all the pathological processes underlying viral myocarditis are initiated by the viral infection. Therefore, it should be of value to patients to receive anti-viral treatment. There is also evidence that early administration of anti-virals can lower mortality and achieve a better prognosis compared with later administration in patients with viral myocarditis caused by H1N1. It is worth noticing that direct injuries caused by viral invasion and replication in the myocardium occur at the early phase of illness and, thus, anti-viral treatment should be started as early as possible.

(1) Drugs such as oseltamivir and peramivir block viral release from cells by inhibiting influenza neuraminidase, thereby suppressing replication and dissemination of the virus. They are effective against Type A and Type B influenza. Oseltamivir phosphate capsules (Duffy) are recommended, 75 mg to be taken orally once or twice a day, as necessary. Peramivir is a neuraminidase inhibitor that can be administered intravenously. Use 0.3–0.6 g $d^{-1}$ i.v. for 3–5 successive days.

(2) Guanylic acid analogues can interfere with and inhibit the synthesis of viral DNA. The most widely used, acyclovir, is effective against DNA viruses such as EBV, while ganciclovir (0.5–0.6 g $d^{-1}$ i.v.) is effective against cytomegalovirus. Since most patients will not have been tested for the exact virus, a combination of these two classes of anti-viral drugs should be considered.

(3) Interferon may be considered, especially when patients are infected with enterovirus (Kühl et al., 2012).

### *Immunomodulating therapy*

Recommendation: All fulminant myocarditis patients should be given immunomodulating therapy by using large dosage of glucocorticoids and intravenous immunoglobulin (IVIG).

The damage caused by fulminant myocarditis is due to direct injuries caused by the virus and indirect lesions caused by the immune system and inflammatory cytokine cascade. Immunomodulating therapy targets the underlying pathophysiology of inflammatory injuries and can help in blocking the onset of illness, reducing inflammation, ameliorating clinical symptoms, rescuing dying myocardium, and, in theory, improving prognosis. Although there are still no clear results from large-scale multi-center clinical studies, the existing results and clinical experiences from our practice strongly suggest its effectiveness and safety; thus, it is recommended.

#### *Glucocorticoid therapy*

Recommendation: Start with 200 mg methylprednisolone i.v. every day for 3–5 days and then reduce dosage according to state of illness.

Glucocorticoids' effects include inhibiting immunological reactions, anti-inflammation, anti-shock, protecting multiple organs from injuries, eliminating allergic reactions, inhibiting inflammatory edema, and reducing the cardiotoxic effects of toxins and inflammatory cytokines. Glucocorticoids should be used at the second phase or the so-called immune injury phase. They should not be used at the first phase during which the virus replicates, causing severe myocardial damage, because glucocorticoids may increase viral replication. However, since the first stage lasts for a short period and immune injuries in the second stage are severe and appear early, it is recommended that an appropriate dose of glucocorticoids can be administered as early as possible. Dexamethasone 10–20 mg can be injected intravenously for fast onset, immediately followed by methylprednisolone 200 mg per day by i.v. drip.

A Cochrane meta-analysis published in 2013 included 719 cases of fulminant myocarditis patients treated with glucocorticoids across eight clinical trials (Chen et al., 2013). The results showed that although there was no difference in mortality rate between the treatment and control groups, the LVEF (Left Ventricle Ejection Fraction) of the patients in the treatment group was higher than that of those in the control group after 1–3 months of follow-up. Importantly, no increases in virus replication or severity of illness took place in the glucocorticoid treatment group, which is strong evidence of its safety. There are only a few case reports and no large-scale clinical trials on using glucocorticoids in the treatment of fulminant myocarditis. The two reports by Bjelakovic demonstrated that large doses of methylprednisolone successfully saved the lives of two children with fulminant myocarditis who had fallen into a state of cardiac shock, metabolic acidosis, hypoxemia, and lactic acidosis, and needed large doses of dopamine and dobutamine that ultimately aggravated their condition (Bjelakovic et al., 2016). They improved after receiving a large dose of methylprednisolone (10 mg $kg^{-1}$ $h^{-1}$). Blood pressure and oxyhemoglobin saturation returned to normal, and left ventricular function recovered totally in 2 weeks. There have also been more studies with relatively large cohorts and similar results from China (Hu et al., 2014; Tang et al., 2014; Wang et al., 2013).

#### *IVIG*

Recommendation: 20–40 g a day for 2 days, and 10–20 g a day for 5–7 days.

IVIG can not only neutralize pathogens such as viruses, but also block the Fc receptor, giving it both anti-viral and anti-inflammatory activity. On the one hand, IVIG can provide the body with passive immunity to help clear viruses. On the other hand, it can modulate the functions of antigen presenting cells and Treg cells, preventing cellular immunity from over-reaction, reducing the attacks of cytotoxic T cells on the myocardium, and reducing cytokine synthesis. As a result, injury to the myocardium can be reduced, left ventricular function can be improved, and severe arrhythmia and death can be avoided.

Although results from large randomized prospective studies are lacking, some small studies revealed that IVIG is beneficial to patients with fulminant myocarditis. An observational study carried out in the United States showed that large doses of IVIG given to patients whose LVEF is less than 30% can increase LVEF from 21.7%±7.5% to 50.3% ±8.6% ($P$=0.005) (Goland et al., 2008). During an average of 13.2 months of follow-up, LVEF was maintained at 53%

±6% and no patients needed rehospitalization. Another controlled study of 21 pediatric fulminant myocarditis patients treated with large doses of IVIG (2 g $kg^{-1}$ in 24 h) revealed that left ventricular end diastolic diameter (LVEDD) in the treatment group improved markedly compared with the control group follow-up (follow-up for 3–6 months, $P$=0.008; follow-up for 6–12 months, $P$=0.072) (Drucker et al., 1994). Recently, a multicenter controlled study of 41 acute myocarditis patients carried out in Japan indicated that large doses of IVIG (1–2 g $kg^{-1}$ for 2 days) can clearly improve one-month mortality rates (20.0% in treatment group vs. 34.6% in control group, $P$<0.01). Furthermore, circulating cytokines such as TNF and IL-6 also decreased significantly ($P$<0.01) (Kishimoto et al., 2014). A retrospective study of 58 fulminant myocarditis patients in Guangdong province of China revealed that compared with patients who did not receive IVIG, patients treated with 400 mg $kg^{-1}$ IVIG had a remarkable improvement in LVEF and LVEDD after 4 weeks ($P$=0.011 and 0.048, respectively). In addition, the incidence rate of ventricular tachycardia or fibrillation decreased significantly in the treatment group ($P$=0.025), and there was a clear tendency toward decreased mortality that did not reach statistical significance (6% vs. 27%, $P$=0.072) (Yu et al., 2014).

Treatment with IVIG should be administered in sufficient doses as early as possible. A retrospective analysis published in 2015 did not report a survival benefit for IVIG use in hospitalized fulminant myocarditis (Isogai et al., 2015). However, on closer examination we find that patients in the treatment group did not receive a sufficiently high dose of IVIG (2 g $kg^{-1}$), which may be the reason for the poor outcomes. In addition, the patients who received IVIG after mechanical circulatory support were included, but those who did not need mechanical circulatory support were excluded. The need for mechanical circulatory support would indicate that the patients were in a critical condition. Thus, administration of IVIG at this time may be too late and incapable of producing positive results. In conclusion, the use of sufficiently high doses of IVIG as early as possible may be the key to all contentions about its effect. High-quality large-sample clinical studies are needed to confirm this.

## *Life support treatment*

Recommendation: All fulminant myocarditis patients should be given life support treatment as early as possible.

Life support is critical to the treatment of fulminant myocarditis and is the center of our "Life Support-Based Comprehensive Treatment Regimen" strategy. The myocardium is diffusely and severely injured and the heart's pump function is dramatically decreased when patients suffer from fulminant myocarditis. Pulmonary congestion and pulmonary inflammatory injury further exacerbate the condition. As a result, blood and oxygen supply to tissues cannot meet the body's demand and consumption. Life support treatment, including circulation and respiration support, can give the heart enough rest, allowing it to regain its normal function under systematic treatment. Therefore, life support treatment ought to be at the center of all treatment plans. Vasopressors, cardiotonics, or catecholamines are the second choice when no life support treatments are available, or they may be used for short periods as preparation for life support treatment.

### *Circulation support*

(1) IABP

Recommendation: All fulminant myocarditis patients with unstable hemodynamics should undergo IABP as soon as possible.

The IABP consists of a catheter with a balloon implanted into the descending aorta, between the distal end of the left subclavian artery and the descending aorta superior to the renal artery. By repeated rhythmical air inflation and deflation of the balloon during diastole and systole, respectively, the IABP can help the heart rest, reduce its load, and provide an auxiliary pump effect. When the balloon is inflated at diastole, it occupies space in the aorta allowing it to increase diastole pressure and increase infusion to the heart, brain, and other tissues. The balloon will deflate just before systole, which lowers the pressure in the aorta, reducing the afterload and work of the heart and increasing stroke volume (SV), forward circulation, and systemic perfusion. The use of IABP in patients with shock can elevate blood pressure, reduce the dose or avoid the use of vasopressor drugs, and help patients survive the acute phase. Our clinical experience, as well as others', proves that fulminant myocarditis patients can significantly benefit from early use of IABP (Ihdayhid et al., 2014; Maisch et al., 2014; Okai et al., 2012).

(2) ECMO

Recommendation: All fulminant myocarditis patients with unstable hemodynamics should receive ECMO as soon as possible if IABP is not enough to recover essential circulation.

ECMO should be used at once if IABP is still unable to sufficiently improve circulation. ECMO can give the heart and lungs enough rest and it is preferably used in combination with IABP. Although fulminant myocarditis patients are more severe than common myocarditis patients, if they can survive the acute phase, their heart function will recover completely and they have good outcomes. Therefore, very critical patients, especially those who suffer from cardiac shock, those whose cardiac index is less than 2 L $min^{-1}$ $m^{-2}$, or those whose blood lactic acid is over 2 mmol $L^{-1}$ can gain more benefit from ECMO. Our experience also demonstrates that the use of ECMO combined with IABP at an early stage of illness can efficiently save patients' lives (Hu et al., 2014;

Yang et al., 2016).

ECMO was first developed in the 1970s. Its mechanism employs a closed system to oxygenate the circulation outside of the body. After dozens of years of improvement, ECMO has evolved into a portable mechanical supportive equipment which is convenient to use, does not need surgery, and can provide long-time life support. The ECMO system mainly consists of three elements: first, a pipe system that draws blood out of circulation and then transfuses it back into circulation; second, a pump (artificial heart) that circulates the blood quickly through the pipe; third, a closed membrane oxygenator that oxygenates the blood (membrane lung). There are several adjunctive pieces of equipment such as thermostatic water tank, the oxygen providing pipe, and several monitoring systems. The working mode includes vein to vein and vein to aorta (VA) modes; the latter should be used for fulminant myocarditis treatment because these patients need circulation support.

The positive effects of ECMO on fulminant myocarditis patients have been proved by several clinical studies. According to previous reports, the median duration of ECMO usage is 5–9 days, and the curative hospital discharge rate is 55%–66% (Diddle et al., 2015; Hsu et al., 2011; Lorusso et al., 2016; Nakamura et al., 2015; Pozzi et al., 2016). A statistical analysis of 3,846 cardiac shock patients who received VA mode ECMO treatment from January of 2003 to December of 2013 revealed that chronic kidney failure, low blood pressure, and low bicarbonate are correlated with a high mortality rate (Schmidt et al., 2015). A retrospective study of patients who received VA mode ECMO treatment shows that predictive factors for poor prognosis are older age, hemorrhage, and multiple organ failures (Nakamura et al., 2015). The predictive potential of cardiac enzymes and BNP is controversial (Hsu et al., 2011; Luyt et al., 2012). Their tendency of returning to normal levels quickly may be of more value than their exact levels (Hsu et al., 2011). In addition, during the treatment of multiple organ failures by ECMO, a decline in liver function (especially when total bilirubin and direct bilirubin markedly rise) is an indication of the deterioration of multiple organ functions and poor prognosis (Diddle et al., 2015). Therefore, some researchers propose that when bilirubin rises quickly or reaches more than 3.0 mg $dL^{-1}$ (1 mg $dL^{-1}$=17.1 μmol $L^{-1}$) during application of ECMO, an LVAD should be employed instead of ECMO (Hu et al., 2014; Nakamura et al., 2015).

*Respiratory support*

Recommendation: All fulminant myocarditis patients should be given respiratory support as early as possible.

Respirator-assisted ventilation can improve pulmonary function and reduce the load on the heart. It is a crucial treatment for patients with fulminant myocarditis complicated with left heart failure, and its use as early as possible is recommended. When patients have symptoms such as tachypnea and labored breathing, respiratory support should be given even if oxyhemoglobin saturation is normal to help reduce the load on the failing heart.

There are two types of respiratory support:

(1) Noninvasive assisted ventilation: It incorporates two working modes: continuous positive airway pressure and duplex intermittent positive airway pressure. Recommended for cooperative patients with tachypnea or whose respiratory rate is over 20 bpm. If it is ineffective or not tolerated by the patient, a tracheal cannula should be used.

(2) Tracheal cannula and artificial controlled mechanical ventilation: This can provide full respiratory support and must be used when patients have fallen into respiratory failure, especially those with obvious respiratory or metabolic acidosis and disturbance of consciousness. It should be used in patients with tachypnea or low oxyhemoglobin saturation who are not responding well to noninvasive assisted ventilation. Its use should also be considered in patients with tachypnea or difficulty in breathing or fatigue who cannot adapt to noninvasive assisted ventilation.

*Hemopurification and continuous renal replacement therapies*

Recommendation: All fulminant myocarditis patients should receive hemopurification therapy as early as possible.

All fulminant myocarditis patients should receive hemopurification therapy as early as possible to filter out toxins and cytokines, regardless of the presence of kidney injuries. Hemopurification therapy can also reduce heart load through ultrafiltration, maintain the water-salt and acid-base balances, and recover the organs' response to treatment drugs. Attention must be paid to the fact that hemopurification needs to be carried out continuously for at least 8–12 h every day in order to eliminate toxins and toxic cytokines, and that blood elicitation at the beginning and retransfusion at the end of hemopurification must be slow to avoid triggering circulatory or heart failures due to the heart's weakened pump function.

Viral infection can activate cellular and humoral immunity, causing monocyte and lymphocyte infiltration, increased translation of cell adhesion molecules, and elevated synthesis of auto-antibodies. These play important roles in the progression of fulminant myocarditis. The persistence of viral infection is central to immune abnormalities, which can eventually lead myocarditis to progress to dilated cardiomyopathy. Therefore, hemopurification therapy is of great importance to fulminant myocarditis patients. Studies reveal that early hemodynamic improvement and decrease of inflammatory injuries can markedly improve prognosis.

Continuous veno-venous hemodiafiltration (CVVHDF) and renal replacement therapies (RRT or CRRT) are widely used in the treatment of heart failure. CRRT uses a blood pump to drive blood from the vein through a filter and then back into the body. It can eliminate cytokines from circulation and help maintain sodium-water balance in a continuous, moderate, and isosmotic fashion. Its functions usually include (i) eliminating different kinds of small molecule toxins and water-soluble inflammatory mediators through convection, diffusion, and absorption, and thereby attenuating inflammatory reactions and organ injuries; (ii) rectifying water-electrolyte and acid-base disturbance, lowering blood temperature, and maintaining homeostasis; (iii) effectively ameliorating tissue edema and improving oxygen delivery and organ functions; and (iv) providing enough liquid intake to enable treatment with other necessary drugs and parenteral nutrition. During CRRT, the variations in blood volume and colloid osmotic pressure will be quite small and tissue perfusion can be maintained at a high level; thus, hemodynamics will not be disturbed.

Although the common indications of CRRTs are oliguria, anuria, hyperkalemia (>6.5 mmol $L^{-1}$), severe metabolic acidosis (pH<7.1), and azotemia (blood urea nitrogen >300 mmol $L^{-1}$), it should be used as early as possible in patients with fulminant myocarditis complicated with acute left heart dysfunction. Circulation failure and shock are not contraindications to CRRT; on the contrary, they are signs of critical illness that necessitates CRRTs. A comparative study between CRRT and diuretic drugs in the treatment of acute heart failure carried out in the United States demonstrated that CRRT can significantly decrease body weight, shorten time spent in the CCU, increase CO and SV, reduce pulmonary capillary wedge pressure, and lower 30-day mortality rate (Badawy and Fahmy, 2012). Studies also reveal that compared with diuretic drugs, CRRT does not significantly interfere with hemodynamic indexes such as heart rate, blood pressure, right atrium pressure, mean pulmonary arterial pressure, systemic vascular resistance, and pulmonary vascular resistance.

Immunoadsorption (IA) therapy is a novel hemopurification technique developed over the past 15 years. Its mechanism combines a ligand (a highly-specific antigen, antibody, or materials with a specific physical or chemical affinity) and a carrier (absorption materials) on an adsorbent reagent or adsorption column. These adsorbent reagents can selectively or specifically eliminate circulating to purify the blood and inhibit disease progression. Since cellular and humoral immunity are involved in the pathophysiological progress of fulminant myocarditis, IA is useful since it selectively targets pathogens and cytokines in plasma. Although there is still a lack of large-scale evidence-based clinical trials, results from small sample clinical studies show that IA therapy can improve heart function, clinical state, indexes of hemodynamics such as cardiac output and stroke volume, systemic vascular resistance, exercise tolerance, and can reduce the levels of NT-proBNP, which are used to evaluate the severity of heart failure (Felix et al., 2015; Jensen and Marchant, 2016). In addition, IA can decrease myocardial inflammatory reactions and improve left ventricle contraction function after usage of protein A immunoadsorption (Bulut et al., 2010). IA therapy can be tried when it is available.

### *Drug therapy of shock and acute left heart failure*

Recommendation: Vasoactive drug therapy can be used temporarily to maintain basic blood pressure.

Fulminant myocarditis is commonly complicated with circulatory shock, and acute left or whole heart failure can be observed in almost every patient. The mechanisms underlying shock involve pump failure, toxemia, and insufficient volume. Most importantly, pump failure distinguishes fulminant myocarditis-complicated shock from other types of shock, and determines the difference in management. Hence, if possible, life support treatment should be administered. If not available, temporary drug therapy is still worthy of consideration.

(1) Drug therapy of shock: Treatments should be taken according to causes of shock. Fulminant myocarditis is often complicated with insufficient volume due to diaphoresis, vomiting and diarrhea, and under this circumstance, appropriate infusion can be considered. The speed and dosage of infusion should be determined by monitoring indexes of hemodynamics. Dopamine and 5% sodium bicarbonate should be used first. Small doses of adrenergic alpha receptor agonist such as aramine can be used whenever it is necessary to maintain the basic vital signs and gain enough time for further treatments. The speed of infusion can never be excessively fast unless there are obvious signs of dehydration. Attention should be paid to the fact that the adrenergic alpha receptor agonists can only be used for a short period. If they are used for long periods, they may aggravate tissue anoxia, do irreversible damage to tissues and organs, and even lead to death. It is noteworthy that administration of dopamine can increase the heart rate, trigger ventricular arrhythmias such as premature beating, ventricular tachycardia, and ventricular fibrillation, and increase cardiac load. Therefore, its dosage should be as small as possible. Glucocorticoids should be used at an early stage as part of an anti-shock therapy.

(2) Drug therapy of acute left heart failure includes positive airway pressure ventilation, hemofiltration, and the use of diuretic drugs. When the heart rate is markedly elevated, a small dose of digitalis can be used. The use of monoamine cardiotonic drug should be avoided for fear of increasing cardiac oxygen consumption and precipitating arrhythmia. Vasodilators should be given with great care because of the risk of low blood pressure. The outflow and intake of fluid should be determined by liquid equilibrium and hemodynamics to prevent the occurrence of acute left heart failure. Life support treatment, including artificial mechanical respiration and circulation as mentioned previously, should be given to severe heart failure and cardiac shock patients to maintain stable hemodynamics and perfusion to important organs, enable the heart to adequately rest, and help patients successfully pass the acute phase.

### *Treatment of arrhythmia*

Recommendation: Administer specific treatment according to the type of arrhythmia and the hemodynamic condition as recommended by guidelines.

Fulminant myocarditis is often complicated by hypotension and shock. If hemodynamics deteriorates due to severe arrhythmia, the patients' lives will be threatened. The management of arrhythmias should follow existing guidance. Full consideration should be given to the heart's pump function and blood pressure and suitable drugs and treatments should be chosen.

Prediction of severe arrhythmia: Sinus bradycardia, prolonged QRS, deterioration of left ventricular function on echocardiography, consistent increases or variability of cardiac troponin, and continuous low perfusion or non-persistent ventricular tachycardia are often signs of severe arrhythmia.

Global guidelines for the treatment of arrhythmia: (i) Quickly diagnose arrhythmias and rectify unstable hemodynamics. Patients with severe hemodynamic dysfunction due to arrhythmia should be given antiarrhythmic treatment immediately. For tachyarrhythmia or ventricular fibrillation, synchronized electric cardioversion or defibrillation should be used at once. Drugs can then be administered if electric cardioversion does not work or arrhythmias reappear after cardioversion. Amiodarone can usually be administered IV with careful consideration of blood pressure. If the arrhythmias cannot be terminated, the first objective should be stabilizing hemodynamics and improving the overall condition. (ii) In patients with relatively stable hemodynamics, the treatment strategy and anti-arrhythmia drugs can be selected according to the clinical symptoms, heart function state, and type of arrhythmia. After successfully correcting arrhythmias, prevention of reoccurrence should be the main objective. (iii) Actively improve cardiac function and rectify hypotension. Deal with disturbances of homeostasis such as electrolyte disturbance and acid-base equilibrium disorders. (iv) Fulminant myocarditis is usually complicated with cardiac dysfunction, cardiac shock, and low perfusion of tissues and organs. Therefore, patients with tachyarrhythmia may not tolerate the negative inotropic effect of negative chronotropic drugs such as beta blockers and non-dihydropyridine calcium channel blockers. Instead, amiodarone should be given through continuous IV pumping, but quick IV injection should be avoided. Patients with atrial fibrillation with a fast ventricular rate can be given digitalis to control the excessively fast ventricular rate. (v) Patients with bradycardia should be implanted with a temporary pacemaker. If temporary pacemakers are not available, drugs such as isoprenaline or atropine can be used to increase the heart rate. Care must be taken when giving isoprenaline because of its arrhythmogenic effects. (vi) Since most patients can recover successfully, permanent pacemaker implantation is not recommended in patients with bradycardia during the acute phase. If the patient's overall state is consistently normal for 2 weeks or longer but a conduction block remains, permanent pacemaker implantation can be considered. Implantation of cardioverter-defibrillator in patients with ventricular tachycardia or fibrillation is not recommended during the acute phase.

All treatment options and their recommendations are summarized in Table 2.

In conclusion, fulminant myocarditis is a specific type of myocarditis characterized by very rapid progress and a very severe state of illness. The hemodynamics of fulminant

**Table 2** Principles and suggestions of treatments to fulminant myocarditis

| Treatment | Suggestion |
|---|---|
| Close monitoring | All fulminant myocarditis patients should be monitored closely. |
| Active symptomatic treatment and supportive treatment | All fulminant myocarditis patients should receive active symptomatic and general management and supportive treatment. |
| Anti-virus therapy | All patients with fulminant myocarditis should receive anti-virus therapy as early as possible. |
| Immunomodulating therapy | All fulminant myocarditis patients should be given immunomodulating therapy including glucocorticoids and IVIG by using large dosage of glucocorticoids and intravenous immunoglobulin (IVIG). |
| Life support treatment | All fulminant myocarditis patients should be given life support treatment as early as possible. |
| Hemopurification and continuous renal replacement therapies (CRRT) | All fulminant myocarditis patients should receive hemopurification therapy as early as possible. |
| Drug therapy of shock and acute left heart failure | Vasoactive drug can be used temporarily to maintain basic blood pressure. |
| Treatments to arrhythmia | Administer specific treatment according to the type of arrhythmia and the hemodynamic condition as recommended by guidelines. |

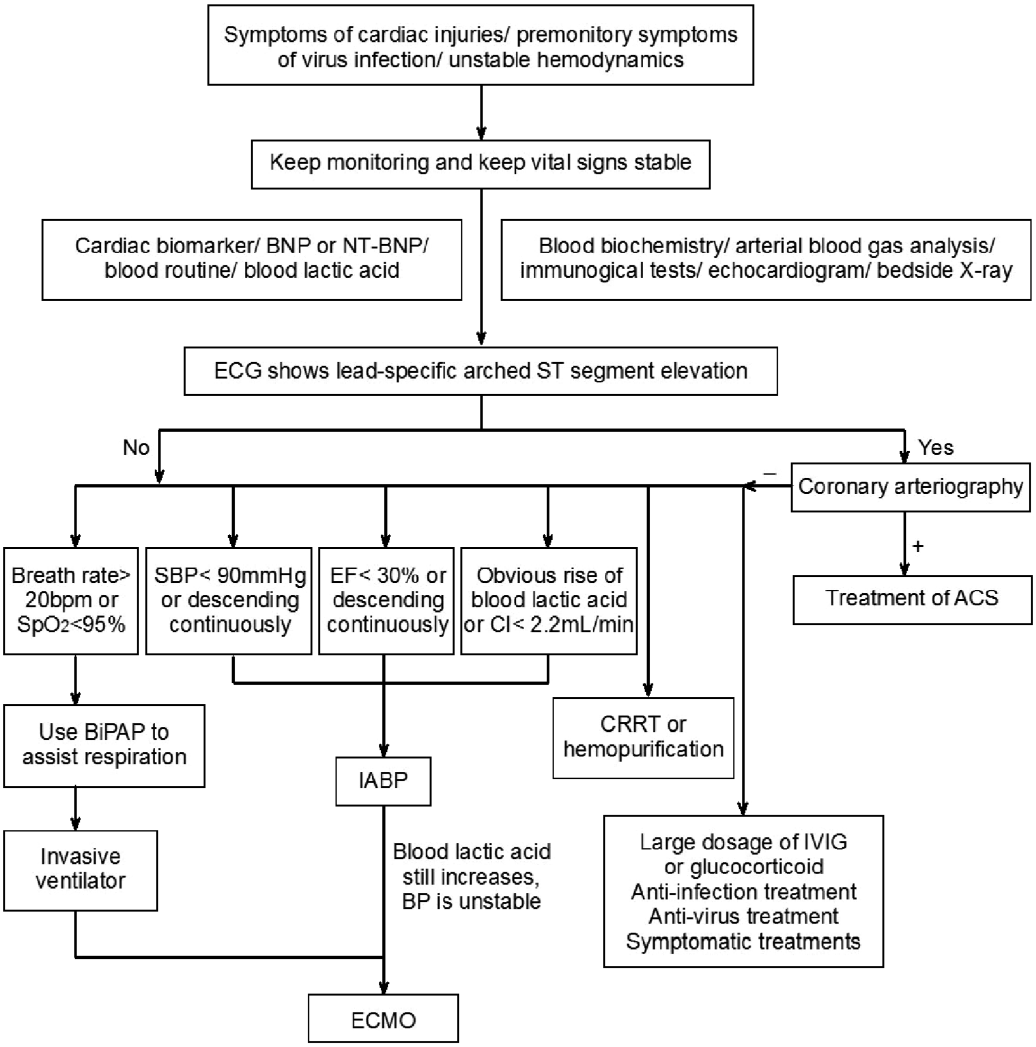

**Figure 1** Flowchart of clinical decision-making process in the treatment of fulminant myocarditis.

myocarditis patients are not stable, and drug treatment cannot achieve good outcomes by itself. In comparison with other severe illnesses, mechanical assisted life support treatments are of great importance to help patients survive the acute phase. Almost all patients can achieve complete recovery if they survive the dangerous acute phase, and if we carefully treat patients while following the "comprehensive treatment regimen based on life support," we can successfully save them. Finally, the authors strongly emphasize the need to make a diagnosis as early as possible, to anticipate and predict the consequences and prognosis of this disease as early as possible, and to start this treatment regimen as early as possible. Never give up on any patient and do everything to save their lives.

Figure 1 summarizes the consensus statement for ease of use during the clinical decision-making process.

**Compliance and ethics** *The author(s) declare that they have no conflict of interest.*

**Acknowledgements** *This project was funded by the National Key Basic Research Project (2012CB518004) and Natural Science Fund Key Project (81630010). All the physicians and nurses of the Department of Cardiology and some of the physicians and nurses of the Department of Nephrology at Tongji Hospital contributed to the treatment of fulminant myocarditis patients and the formulation of this consensus. Prof. Rutai Hui in Fuwai Hospital gave enthusiastic support and valuable advice to this project. We thank greatly all senior experts for repeated discussion for this expert consensus statement: Jun Pu (Department of Cardiology, Renji Hospital, School of Medicine, Shanghai Jiao Tong University), Yundai Chen (Department of Cardiology, Chinese PLA General Hospital), Yugang Dong (Department of Cardiology, the First Affiliated Hospital of Sun Yat-Sen University), Weiyi Fang (Department of Cardiology, Shanghai Chest Hospital, Shanghai Jiao Tong University), Junbo Ge, Jingmin Zhou (Shanghai Institute of Cardiovascular Diseases, Zhongshan Hospital, Fudan University), Xianghua Fu (Department of Cardiology, the Second Affiliated Hospital of Hebei Medical University), Yong Huo (Department of Cardi-*

*ology, Peking University First Hospital), Rutai Hui (Department of Cardiology, Fuwai Hospital), Jianjun Tan (Department of Cardiology, the Second Affiliated Hospital of Xiangya School of Medicine, Central South University), Lemin Wang (Department of Cardiology, Tongji Hospital, Tongji University School of Medicine), Zonggui Wu (Department of Cardiology, Changzheng Hospital, the Second Military Medical University), Dingli Xu (Department of Cardiology, Nanfang Hospital, Southern Medical University), Dingcheng Xiang (Department of Cardiology, Guangzhou General Hospital of Guangzhou Military Area Command of Chinese PLA), Shenghua Zhou, Tianlun Yang (Department of Cardiology, Xiangya Hospital of Central South University), Bo Yu (Department of Cardiology, 2nd Affiliated Hospital of Harbin Medical University), Jing Zhang (Fuwai Huazhong Cardiovascular Hospital) and Song Zhang (Cardiovascular Department, Xin Hua Hospital, Affiliated Hospital of Shanghai Jiao Tong University).*
